# Chocolate Milk versus carbohydrate supplements in adolescent athletes: a field based study

**DOI:** 10.1186/s12970-019-0272-0

**Published:** 2019-02-12

**Authors:** Katelyn A. Born, Erin E. Dooley, P. Andy Cheshire, Lauren E. McGill, Jonathon M. Cosgrove, John L. Ivy, John B. Bartholomew

**Affiliations:** Department of Kinesiology and Health Education, The University of Texas at Austin, 2109 San Jacinto Blvd, Mail Stop D3700, Austin, TX 78712-1204 USA

**Keywords:** Post-exercise supplements, Chocolate milk, Strength, Adolescents, Field-based, Translational research

## Abstract

**Purpose:**

The purpose of this study is to translate laboratory-based research on beverage-based supplements to a naturalistic, field setting in adolescent athletes. To this end, we tested the effects of two commercially-available drinks on strength in a field-based setting with both male and female high school athletes completing a summer training program.

**Methods:**

One hundred and three high school athletes completed the study (*M* age = 15.3, *SD* = 1.2; 70.9% male; 37.9% Afr. Amer.). Measures included a composite strength score (bench press + squat). Participants completed 1 week of pre- and post-testing, and 4 days per week of strength and conditioning training for 5 weeks. Participants were randomly-assigned to receive either CM or CHO immediately post-exercise.

**Results:**

A 2 (group) × 2 (time) repeated measures ANOVA showed there was a significant main effect on time for increase in the composite strength score (*p* = .002, *ŋp2* = .18). There was a significant interaction of composite strength score between groups, (*p* = .04, *ŋp2* = .08). The CM group (12.3% increase) had significantly greater improvements in composite strength from pre- to post-test than CHO (2.7% increase). There were no differences in these results based on demographic variables.

**Conclusion:**

This is the first study comparing the impact of CM and CHO on athletic outcomes in an adolescent population in a field-based environment. CM had a more positive effect on strength development and should be considered an appropriate post-exercise recovery supplement for adolescents. Future research will benefit from longer study durations with larger numbers of participants.

## Introduction

Resistance exercise results in several adaptations to the musculoskeletal system. Specifically, it results in greater strength and hypertrophy [1]. These effects are especially potent in untrained individuals [2]. Additionally, resistance exercise plays a significant role in athletic performance, injury prevention [3], quality of life [4], and lowering the risk of all-cause mortality [5]. Given the implications for both performance and health, much of the recent literature explores methods to optimize these training effects.

One approach to improve the response to resistance training is the use of post-exercise nutritional interventions; with carbohydrate-protein (CHO + PRO) beverages garnering significant interest. Much of the work has been applied to endurance training, where CHO + PRO supplements have been shown to be the superior post-exercise recovery compared to a carbohydrate (CHO) only supplement in 32 untrained male and female participants (18–35 years old) and in eight untrained males (*M* age = 24) [6, 7]. While promising, these data cannot be directly applied to resistance exercise as there are unique challenges to supplementation. There is some evidence that resistance exercise results in an increase in cortisol levels [8]. Additionally, resistance training has shown to have no effect or a negative effect on insulin levels in male strength athletes (*M* age = 27) [9]. This may be problematic for protein accretion because cortisol increases the rate of protein degradation compared to synthesis and therefore leads to a catabolic state [10] and insulin has been shown to block protein degradation [11]. As such, it may be that the optimal supplement should both attenuate cortisol levels and increase insulin levels.

CHO increases insulin helping to attenuate the post exercise cortisol response. In combination with the anabolic response to protein supplementation, this provides a net positive influence on protein synthesis. Additionally, the attenuation of the cortisol response has been shown to be greatest when CHO and PRO are combined, compared to CHO or PRO alone in a sample of untrained young adult males [8]. Given this positive impact on the anabolic response, it is not surprising that a recent systematic review has found studies consistently show an increase in muscle mass and strength with CHO + PRO supplements compared to CHO or PRO alone supplements in healthy adults [12]. For example, one study investigated the effects of post-exercise supplements during a 12-week resistance training program where participants were untrained young adult males (*M* age = 21) who trained two times a week and ingested liquid supplements during and following workouts. Participants were randomly assigned to a CHO + PRO, CHO alone, or placebo drink. The CHO + PRO group had the greatest gains in strength with significant increases in muscle mass and cross-sectional area (20–27%) compared to the CHO (14–18%) and placebo group (7–9%) [8].

One of the challenges of translating this research into practice is the use of controlled ratios of CHO to PRO within supplementation. Ratios that have been used in the laboratory are difficult to find in quantity and at acceptable cost within the general population. In light of this, chocolate milk (CM) represents a readily-available and cost-effective CHO + PRO food that could replace supplements. The rationale for CM as a sports-recovery drink centers on its nutrient profile. A 250 mL serving of low-fat (2%) CM provides 12 g of CHO as lactose, approximately 8–12 g of sucrose and cocoa, and (depending on the manufacturer) has naturally high levels of electrolytes [13]. This combination presents the consumer with an 8–9.6% CHO solution, which is within the range of optimal CHO density to avoid impaired gastric emptying [14, 15] and enhances glycogen repletion post-exercise [16, 17]. Additionally, CM provides 1 g of PRO per fluid ounce (31.25 mL) with a CHO to protein ratio of 3:1 [13] which has been associated with muscle hypertrophy [18, 19]. In addition, milk may provide an important source of electrolytes, which have been shown to benefit fluid recovery following exercise [13]. One study specifically tested the differences between milk, milk with NaCl (20 mmol/L), a sports drink, and water following an exercise-induced hypohydration protocol. Results indicated that the sports drink (1205 mL) and water (1184 mL) groups excreted twice as much fluid after 1 h compared to both milk groups (611 and 550 mL) [15]. Milk may be the superior option for retaining fluid and therefore a preferred recovery drink. The combined factors of CM’s nutrient profile promote the drink as an excellent post-exercise supplement for replenishing muscle glycogen, building muscle, and re-establishing positive fluid balance. Additionally, CM provides a low-cost, readily available option when compared to other supplements that may provide the same physiological impacts.

There are, however, a number of limitations to the existing research. First, the studies have been primarily limited to highly controlled laboratory settings [6–9, 13]. While the control exhibited in the lab removes confounders and maximizes the ability to infer causality, the sample sizes are typically small (6–32) and have been generally done in young, untrained healthy adult populations, and the constraints on participants do not match common modes of training and supplementation. As a result, these efficacy studies suffer from limitations to generalizability that undermine the ability to translate this work to practice. In addition, there are no studies investigating the effects of post-exercise nutritional interventions in an adolescent population. A recent survey by the National Federation of State High School Associations has found that 7,963,535 adolescents played high school sports in the 2016–2017 season [20]. This represents a large population that has been ignored in field-based, post-exercise nutrition interventions. Chocolate milk has been shown in laboratory research to improve strength in young untrained males [8], yet has not been studied as a low-cost, readily available post-recovery drink that can be implemented on a youth population level. As such, the purpose of this study is to translate these laboratory-based studies on beverage-based supplements to a naturalistic, field setting in adolescent athletes. To achieve this aim, we tested the effects of two commercially-available drinks (CM as the CHO + PRO supplement, and a sports drink as the CHO only supplement) in a field-based setting with both male and female high school athletes who were completing a summer training program. Based on the results of previous research in young adults, we hypothesize that the CM group would experience greater increases in strength compared to the CHO group. These outcomes were expected to be similar for males and females.

## Methods

### A priori power calculation

The G*Power software developed by Faul and Erdfelder [21] was used to determine a priori sample size. The following parameters were selected: moderate effect size (*f* = .25) as seen in the literature [12], α level of .05, a power level of .9, four groups, two measurements, a correlation among the repeated measures of .5, and one for the nonsphericity correction (€) for the RM ANOVA within-between design. The sample size was determined to be at least 46 total.

### Participants

Participants were recruited from a seven-week, summer strength and speed camp at a large southwestern high school. Inclusion criteria included: English speaking individuals who had a cellphone with texting capabilities, free from injuries barring them from physical training, other contraindicated physical or mental disabilities, no lactose intolerance, and no food allergies related to the provided drinks. Based on these criteria, three participants were excluded due to lactose intolerance. Participants were randomly assigned to either CM (CHO + PRO) or CHO by age, sex, grade, and skill level. One-hundred and thirty-one participants enrolled in the study. Twenty-eight participants did not complete the study resulting in a final sample of 103 – a 78.6% retention rate. Approval for the study was obtained from the Institutional Review Board (IRB) at The University of Texas at Austin. Before beginning, all participants provided written informed consent. For those under 18 years of age their parent/legal guardian also provided written consent to participate. This study was funded by Dairy MAX Incorporated.

### Research design

This study was a 2 (group) × 2 (time) mixed factorial design. The training protocol lasted 7 weeks and included: baseline testing during week 1, 5 weeks of training, and post-testing during week 7. Baseline and post-test measures included height, body weight, bench press, squat, and power clean.

### Protocol

In an attempt to achieve a naturalistic setting, this study took advantage of a pre-existing, seven-week high school strength and speed camp that was open to all students during the summer. Athletic coaches at the same large public high school implemented the camp and participants paid $150 to the athletic program to participate. Those who were eligible for free and reduced lunch were provided a waiver to participate at no cost. Participants completed the consent form and a demographic information survey (race, ethnicity, sex, age, year in school, and sport) prior to the start of the study. The coaching staff did not agree to release data related to socio-economic status. Overall at the school-level, 26.2% of the high school’s students were economically disadvantaged, 14.1% were African American, 37.2% were Hispanic, 37.6% were white, 0.4% were American Indian, 5.8% were Asian, 0.3% were Pacific Islander, and 4.6% were two or more races for the 2016–2017 school year.

Participants were placed into three training groups by the athletic coaching staff depending on sex and age. The first group consisted of both junior varsity (JV) and varsity female athletes entering grades nine through twelve (*n* = 30). The second group consisted of varsity male athletes entering grades eleven and twelve (*n* = 43). The third group consisted of junior varsity male athletes entering grades nine and ten (n = 30). The summer camp was held 4 days a week, Monday through Thursday. Each day consisted of a 1-h free weight resistance training session led by the high school’s strength coach followed by 1-h of on-field agility drills and conditioning sprints led by the athletic coaches. Each of the 4 days contained a different set of strength and speed exercises. These exercises switched halfway through the camp, so a new set of exercises started during week four. Start times for each group were staggered by 1 hour. As such, when group one completed the resistance training and moved to the agility and conditioning training, group two began their resistance training. Group three began their resistance training once group two moved to their agility and conditioning training. After completion of the full, 2-h training session, participants were provided with one dose of their assigned supplement that they consumed in the presence of research staff immediately post-exercise. They were instructed to not ingest anything other than water during training and until 1 hour following consumption of the supplement. Otherwise, they could eat and drink ad libitum.

Strength pre-tests were scheduled by the coaching staff. The girls and JV boys were tested during the first week of training as part of their scheduled workouts. Pre-tests for the varsity boys occurred during the last week of May, 2 weeks before the start of the study. Post-tests for all three groups were conducted by the coaching staff during the last week of training, at the beginning of their normally scheduled weight training time. Strength assessments for bench press and squats were conducted on subsequent days – bench press was completed on a Monday and squat was completed on a Tuesday for both pre- and post-testing. Weight and height measurements were completed prior to training during the first week of training for the pre-test and during the last week of training for post-test.

### Experimental beverages

Chocolate milk (CM) (*Horizon Organic Low-Fat Chocolate Milk, WhiteWave Foods Company, Denver, CO, USA*) was used as the CHO + PRO supplement. A commercially available sports drink was used as the CHO supplement. The kcal and macronutrient composition of the beverages are shown in Table 1. The amount of supplement was not stratified by age, weight, or sex. The purpose of this study was to allow for high school athletes to achieve the same benefits of post-exercise supplements that were readily available in a low-cost single-serving portions. The drinks were chosen to match CHO content. Although not perfectly matched for fluid or kcals, the drinks were commercially available and therefore have real world value. No two CM or CHO drinks came with the same milliliter per unit. Therefore, researchers would have to pour out at least one of the beverages and add calories to the drink to match liquid quantity and kcals and thus would not be considered a practical option in the future for adolescent athletes.

**Table 1:** Kilocalorie and macronutrient composition of the supplements

	CM	CHO
Volume (mL)	473.1	709.7
CHO (g)	44	42
PRO (g)	16	0
Fat (g)	5	0
Sodium (Na) (mg)	360	320
Kcal	300	160

*Abbreviations*: *CHO* Carbohydrate, *PRO* Protein, *CM* Chocolate Milk, *g* gram, *mg* milligram, *Kcal* Total kilocalories

### Measures

#### Height

Height was self-reported to the nearest centimeter (cm). To reduce participant burden, and to not impede on their training, the researchers chose to use the convenience and speed of self-reported height.

#### Body weight

Body weight was measured using the Tanita BWB-800 scale (*Tanita Corporation of America, Arlington Heights, IL, USA*). Weight was measured to the nearest 0.1 kg (kg). The scale was calibrated before each trial. Participants’ weights were measured in workout clothes and without shoes. Weight was measured twice for each participant, and an optional third measurement was taken if the two measurements differed by 0.1 kg.

#### Strength assessments

Participants in the female and 9th–10th grade male groups completed a three-repetition maximum for the bench press, squat, and power clean. Participants were instructed to lift as much as possible through a full range-of-motion three times in a row. If they could lift the bar beyond three repetitions, they stopped and progressively increased the weight until they reached a load they could not lift more than 3 times. The 11th–12th grade male athletes followed an identical protocol to achieve a load they could not lift beyond 1-repetition. The difference in protocol was in-place as a part of the camp. As the 11th–12th grade males had more experience with lifting, the coaches believed them to be at lower risk of injury and more likely to achieve an accurate 1-repitition maximum than the other groups. As the existing camp procedures were utilized, the difference in measures was maintained but sub-group analyses were done to compare the groups. Since the camp was designed to target both upper and lower body for strength, a composite strength measure was made that combined bench press and squat, as an indicator for a full body strength. After observing the baseline measures it was clear that the complexity of the power clean lift was beyond the skill of most participants and the research team was not confident in the resulting data. Therefore, this measurement was not used as an outcome.

### Statistical analyses

Changes in body weight, bench press, squat, and a composite strength score (combined bench press and squat) from pre-test to post-test were analyzed using separate 2 (group) × 2 (time) repeated measures analysis of variance (RM ANOVA) due to differences in the number of participants who completed each measure. Differences were considered significant at *p* < .05. Effect sizes were calculated using partial eta squared (*ŋp*^*2*^) for the RM ANOVA. All statistical analyses were performed using SPSS version 22.0 statistical software (*SPSS Inc., Chicago, Ill, USA*).

## Results

### Participants

One hundred and three adolescent athletes (*M* age = 15.3, *SD* = 1.2; 70.9% male; 37.9% African American) completed the study. Participant characteristics appear in Table 2. Chi-square analyses revealed no difference in sex (*p* = .79), race (*p* = .45) or ethnicity (*p* = 58) between groups. Additionally, independent samples t-test revealed no differences in groups in age (*p* = .16), initial weight (*p* = .12) or height (*p* = .48). Thus all groups were analyzed together.

**Table 2:** Subject characteristics at baseline

	All subjects (*N* = 103)	Male varsity subjects (n = 43)	Male JV subjects (*n* = 30)	Female subjects (n = 30)
Age in years (Mean ± SD)	15.3 ± 1.2	16.2 ± 0.93	14.3 ± 0.55	14.90 ± 1.2
Weight in kg (Mean ± SD)	73.4 ± 18.6	83.9 ± 18.0	66.6 ± 14.4	65.2 ± 15.9
Height in cm (Mean ± SD)	172.3 ± 8.9	177.6 ± 5.6	170.7 ± 9.3	165.7 ± 7.7
Race n (%)				
African American or Black	39 (37.9)	22 (51.2)	5 (16.7)	18 (60)
White	33 (32)	6 (14)	15 (50)	6 (20)
Asian	2 (1.9)	1 (2.3)	1 (3.3)	1 (3.3)
Other	24 (27.4)	13 (30)	9 (30)	5 (16.3)
Ethnicity n (%)				
Hispanic or Latino	26 (25.2)	6 (14)	16 (53.3)	7 (23.3)
Not Hispanic or Latino	75 (72.8)	36 (83.7)	13 (43.3)	23 (76.7)
Sex n (%)				
Male	73 (70.9)	43 (100)	30 (100)	0 (0)
Female	30 (29.1)	0 (0)	0 (0)	30 (100)

### Body weight

There was no significant main effect for changes in body weight, *F*(1, 101) = 3.34, *p* = .07 across time. Additionally, there were no significant interactions between groups in body weight *F*(1, 95) = .49, *p* = .43, *ŋp2* = .01 across time. There were no effects of demographic variables on group differences. These results can be found in Table 3.

**Table 3:** Body weight, body composition, and strength assessments

	Overall	CM	CHO	Main effect				Interaction	
	Mean ± SD	Mean ± SD	Mean ± SD	F (df)	*p*-value	ES	F(df)	p-value	ES
Body weight (kg)									
Pre	73.3 ± 18.5	70.3 ± 16.7	76.8 ± 19.8	3.34 (1, 101)	0.07	0.03	0.49 (1, 101)	0.43	0.01
Post	74.4 ± 18.8	71.0 ± 16.6	77.4 ± 20.3						
Composite Strength Score (kg)									
Pre	173.6 ± 81.9	166.8 ± 73.5	179.7 ± 89.5	11.09 (1, 49)	0.002	0.18	4.3 (1, 49)	0.04	0.08
Post	185.8 ± 80.2	187.3 ± 75.6	184.5 ± 85.5						
Bench press (kg)									
Pre	73.1 ± 32.5	71.8 ± 31.8	74.5 ± 33.7	.01 (1, 79)	0.94	<.001	5.01 (1,79)	0.03	0.06
Post	73.3 ± 31.2	74.3 ± 29.4	72.2 ± 33.5						
Squat (kg)									
Pre	104.4 ± 51.2	100.1 ± 43.1	108.5 ± 58.2	14.5 (1, 56)	<.001	0.21	1.07 (1, 56)	0.31	0.002
Post	116.2 ± 50.8	115.2 ± 46.6	117.1 ± 55.1						

*Abbreviations*: *CHO* Carbohydrate drink, *CM* Chocolate Milk drink, *SD* Standard deviation, *kg* Kilogram

### Strength assessments

These results can be found in Table 3. The composite strength score showed a significant main effect of time, *F*(1, 49) = 11.09, *p* = .002, *ŋp2* = .18, that was qualified by a significant interaction between groups across time, *F*(1, 49) = 4.3, *p* = .04, *ŋp2* = .08. Specifically, the CM group had a greater improvement in composite strength score from pre-test (*M* = 166.8, *SD* = 73.5) to post-test (*M* = 187.3, *SD* = 75.6) compared to the CHO group pre-test (*M* = 179.7, *SD* = 89.5) to post-test (*M* = 184.5, *SD* = 85.5). To better understand this effect, we examined the specific effects on the bench press and squat. There was no significant main effect for changes in bench press across time, *F*(1, 79) = .01, *p* = .94, *ŋp2* < .001. However, there was a significant interaction for bench press between groups across time, *F*(1, 79) = 5.01, *p* = .03, *ŋp2* = .06. Specifically, the CM group increased their bench from pre-test to post-test while the CHO group decreased their bench from pre-test to post-test. There was a significant main effect for changes in squat score across time, *F*(1, 56) = 14.5, *p* < .001, *ŋp2* = .21. The predicted interaction between groups across time was not significant, *F*(1, 56) = 1.07, *p* = .31, *ŋp2* = .02. The effect sizes for each of the two sub-groups were analyzed. These data indicated that both groups increased their squat score from pre- to post-test, with CM, *d* = .34 and CHO, *d* = .15. Thus, while not a significant interaction, the effect sizes trend in the predicted pattern. There were no effects of age, race, or ethnicity on group differences.

## Discussion

This study was designed to translate laboratory-based research on beverage-based supplements to a naturalistic, field setting in adolescent athletes. As such, it is the first to test the effectiveness of a commercially available form of CM as a post-exercise recovery method on strength in adolescents in a field trial in comparison to the effects of commercially available sports (CHO) drink. The results indicated that the CM group had greater improvements in the composite strength score compared to the CHO group.

The improvements in strength due to CHO + PRO and CM use have been found in previous studies. In one study of young (18–25 years old) healthy untrained males, the effects of fat-free milk, soy milk, and a CHO only drink were used to determine the effects of milk versus soy protein on adaptations to resistance exercise. They found that the fat-free milk group had the greatest increase in Type II muscle fiber area compared to both the soy and CHO group [22]. Additionally, there were trends toward significant differences among groups for strength. It is notable that these trends were found in a previous study that showed greater improvement in strength for CHO + PRO compared to CHO, but not at a statistically significant level [8]. This mirrors our findings for the squat. In contrast, this is the first study to show a *decrease* in bench press strength after 5 weeks of training for CHO group compared to an *increase* in strength in the CM group. One possibility is that the participants in the CHO alone condition were less motivated for the post-test. However, we consider this unlikely. Because the participants were athletes, being trained and assessed by their coaches, there was great incentive to provide maximal effort. An alternative explanation for these data is that the high intensity training sessions coupled with an insufficient post-exercise recovery method that includes protein, prevented proper recovery that may have resulted in decreased strength. Therefore, the observed effect may be due to the CHO + PRO group being able to better recover from the training load compared to the CHO group. This is plausible given what is generally known about the benefit of CHO + PRO over CHO alone during recovery that has been elucidated in a review on these effects in females [23]. Relatedly, one study found that participants who consumed CHO + PRO within 2 h post-exercise had better recovery 24 h following an exhaustive bout of cycling than those who consumed an isocaloric CHO beverage in endurance trained males [7]. The ability to recover from exercise is necessary to increase strength during training. Similar findings for the benefits of CHO + PRO over CHO alone have been found for recovery at 4 hours [6] and 12–15 h post exercise [24]. Future research might consider an assessment of daily recovery from strenuous activity and if CHO + PRO use versus CHO alone allows individuals to train at a higher cumulative intensity.

The primary limitations to translational research – especially those that are not structured as randomized control trials – are the threats to internal validity. This study is no different. The outcome measures and training protocol were designed and implemented by the high school coaches, which lead to different timing for strength-testing protocols between the training groups. While the impact is likely minimal as the participants were randomly assigned to condition within these groups, we cannot rule out the potential of this timing to interact with condition. Likewise, it is far more difficult to control behavior under these conditions. For example, it is largely impossible to ensure consistent effort throughout training and to control diet and hydration outside of the supplements. The athletes were asked to not consume any food or supplements up to an hour post workout. However, data were not collected to check if athletes complied with this directive. Additionally, this study did not track the weight concerns of the athletes or if they were using other protein supplements to gain lean muscle mass. Each of these could impact other behaviors that are tied to change in muscle mass and constitute a major limitation. These limitations are off-set by the randomization to condition and the assumption that these issues would be equally distributed across conditions. That said, this lack of control certainly increases variance in response to treatment in a way that makes it much more difficult to detect a significant effect of either condition. An additional major limitation is that the beverages were not matched for calories. The literature shows a tension between post recovery drinks and the amount of carbohydrate in these drinks. It was a long-standing belief that the carbohydrate content in recovery drinks were needed in order to replenish the glycogen stores within muscle. We wanted to extend the research that shows when carbohydrate is paired with protein is when you have maximum effects. Additionally, we wanted a practical supplement option, one where athletes can just go in to the store and buy it, with no hassle of measuring things out. Therefore, we chose two drinks that were commercially available that matched for carbohydrate content.

While clear limitations exist, this design does increase the ability to generalize these results and provides a more reasonable estimate of the effect that would occur if a high school team were to adopt regular supplementation with CM or CHO beverages post workouts. The summer program used for this study is similar in design and activities as those used in many settings and included a highly diverse sample of athletes. Our data do show that high school athletes are willing to drink a volume of CM or sports drink that has been shown in laboratory settings to have an impact post exercise and that CM has positive effects compared to a CHO beverage alone in this sample.

These limitations provide a number of lessons for future research in the high school athletic setting. First, the use of existing programs does not lessen the need for planning and working with local partners in the design stages of the project. Our partner high school was one of the largest and more successful athletic programs in Texas. Moreover, the majority of the athletes had spent at least 1 year training with the coaching staff. As such, we assumed that the athletes were reasonably experienced and skilled with the lifts being performed. While this was a safe assumption for the bench press and squat, the athletes could not adequately perform power cleans. Had we spent time observing training prior to the study we would have modified our procedures prior to study onset (either removing power cleans or providing additional training). We instead focused planning on visits to develop procedures for storing and distributing the beverages. While this was a critical effort for a smooth implementation, future research should be sure to work with their partners to pre-assess all aspects of the design and outcome measures. A more positive lesson for planning to study high school athletes was the coaches’ willingness to provide access as long as the design did not significantly modify their practices and the interventions were sustainable. Finally, it is important to recognize that there are significant barriers to research with high school athletes regarding obtaining district, school, and parent support, but these can be overcome with sufficient preparation. We began the process of recruiting the district and school more than 1 year prior to the study. In addition, as the majority of high school students are minors, their participation required active, parental consent. We began reaching out to parents more than a month prior to the onset of the study. Thus, sufficient lead time is critical for successful implementation of translational studies in the high school athletic setting. In summary, the use of intact, high school athletic teams in the United States is far more viable than the lack of research would suggest. It requires more planning and patient work with local partners than is required for laboratory studies, but given the levels of participation in high school sports, this is a population that requires more study and justifies this effort.

## Conclusion

Research with nutritional supplements are designed to inform practice. The effects of various post-exercise recovery drinks have been well established in laboratory settings, but there was a clear need for translational research to test the effectiveness and feasibility of these beverages in a real-life setting and with an adolescent population. The present study was the first to test the feasibility and impact of CM in this kind of field-based setting with high school athletes. This study shows the importance of working with partners (high school coaches) during the planning stages of studies and training programs. The lack of control of the athletes’ diet and testing procedures were threats to internal validity, but the positive results inform the practice of these athletes. The study also guides the design of future research as it highlights the challenges and potential of these field-based designs.

## Abbreviations

CHO + PRO: Carbohydrate-protein
CHO: Carbohydrate
cm: Centimeter
CM: Chocolate milk
g: Gram
IRB: Institutional Review Board
JV: Junior varsity
kg: Kilogram
M: Mean
mL: Milliliter
mmol/L: Millimoles per liter
ŋp2: Partial eta squared
RM ANOVA: Repeated Measure analysis of variance
SD: Standard deviation
